# Global scientific trends in hypercholesterolemia research from 2003 to 2023: a data-driven bibliometric and visual analysis

**DOI:** 10.3389/fcvm.2025.1524697

**Published:** 2025-06-11

**Authors:** Haoxin Guo, Qihui Wang, Yinling Wang, Xiaolong Dong, Zhongqing Wang, Hui Kang, Shitong Cheng

**Affiliations:** ^1^ Department of Information Center, The First Hospital of China Medical University, Shenyang, China; ^2^ School of Health Management, China Medical University, Shenyang, China; ^3^ National Clinical Research Center for Laboratory Medicine, Department of Laboratory Medicine, The First Hospital of China Medical University, Shenyang, China; ^4^ Laboratory Medicine Innovation Unit, Chinese Academy of Medical Sciences, Shenyang, China

**Keywords:** hypercholesterolemia, familial hypercholesterolemia, cardiovascular disease, bibliometric analysis, VOSviewer

## Abstract

**Background:**

Cardiovascular disease remains the leading cause of mortality and disability worldwide. Hypercholesterolemia is a significant causal factor of ischemic heart disease, contributing to half of all cardiovascular fatalities.

**Methods:**

This study utilized bibliometric tools to offer a comprehensive overview of the current research trends in hypercholesterolemia. The full records and cited references from 18,641 publications (2003–2023) were retrieved from the Web of Science Core Collection, and bibliometric analysis was conducted using VOSviewer.

**Results:**

The United States and Harvard University had the most significant influence among the countries/regions and research institutions, respectively. Among the researchers, Kastelein J.J.P. published the highest number of related articles, whereas publications by Sabatine M.S. had the highest average citation. The top 10 keywords were atherosclerosis, familial hypercholesterolemia, cardiovascular disease, risk, risk factors, gene expression, coronary heart disease, low-density lipoprotein, statins, and prevalence. These high-frequency keywords were clustered into groups based on the pathogenic mechanisms, disease prevalence and prevention, drugs and treatments, and familial hypercholesterolemia. These clusters denote distinct study fields and current research hotspots for hypercholesterolemia.

**Conclusion:**

Through bibliometric and visual analysis, this study comprehensively assessed global research trends and focal areas within hypercholesterolemia, offering valuable insights into current and future research directions in the field. Further research is needed on the relationship between the intestinal microbiota and cholesterol metabolism and on the advancement of microbiota therapy and precision medicine.

## 1 Introduction

Despite advancements in global health, cardiovascular disease (CVD) remains the leading cause of mortality and disability worldwide, and its incidence steadily increases every year (1). The World Health Organization estimates that 17.9 million individuals succumb to CVDs annually, accounting for approximately one-third of all global deaths, which is equivalent to one fatality every 1.75 s. More than three-quarters of these CVD-related deaths occur in low-income and middle-income countries (2). Among the 17 million premature deaths due to non-communicable diseases in individuals aged under 70 years in 2012, 38% were attributed to CVDs (2). Ischemic heart disease comprises half of these CVD fatalities, with hypercholesterolemia serving as a major causative factor (3).

Hypercholesterolemia is characterized by elevated plasma levels of low-density lipoprotein cholesterol (LDL-C), which plays a pivotal role in atherosclerosis development and directly correlates with the risk of atherosclerotic CVD and ischemic heart disease (4). The accumulation of cholesterol-rich LDL over time fosters the formation of fat-containing foam cells and proliferation of atherosclerotic lesions, thereby heightening the CVD risk (5). Individuals with hypercholesterolemia have been shown to face more than a twofold higher risk of CVD (6). Existing evidence confirms that lowering plasma LDL-C levels effectively diminishes the CVD risk and remains integral to CVD prevention. Consequently, most current guidelines for dyslipidaemia management or CVD prevention prioritize LDL-C as the primary treatment target (5). The strategy of lowering LDL-C levels to prevent primary and secondary coronary events has been one of the most crucial achievements in medicine over the past 30 years (7).

The mechanisms, intervention targets, prevention, and management strategies for hypercholesterolemia have captivated the majority of medical researchers, leading to a significant surge in published articles. However, amidst this vast literature, researchers face challenges in pinpointing high-quality papers (8). Bibliometrics, an informatics branch, delves into the literature system and the characteristics of bibliometrics by employing quantitative and qualitative analyses of documents. This method quantitatively assesses the profile distribution, relationships, and research field clustering, serving as a popular technique for assessing the credibility, quality, and impact of academic work (9). Tools such as VOSviewer and CiteSpace, commonly used for bibliometric visualization, facilitate data analysis and visualization (10, 11) and effectively evaluate the thematic development of structured content, thus aiding readers' intuitive comprehension (9).

To date, there has been no bibliometric analysis of hypercholesterolemia despite mounting public health concerns, an increase in published records, and a growing body of scientific evidence. Therefore, this study aimed to utilize bibliometric tools to scrutinize publishing trends in hypercholesterolemia research over the past 20 years. The objectives included identifying influential journals, countries, institutions, and authors; exploring international collaboration networks; uncovering research hotspots and emerging topics; and presenting readers with a comprehensive overview of current hypercholesterolemia research.

## 2 Methods

### 2.1 Source database

For this study, all literature data on hypercholesterolemia were sourced from the Web of Science Core Collection (WoSCC). The WoSCC database is updated continuously, dynamically, and rigorously to assess all publications, making it ideal for our bibliometric analyses. We conducted a literature analysis using the WoSCC to gather general information on the publication year, journal, organization, author, and research area distribution. The literature search was conducted on 25 February 2024, and all bibliometric data were downloaded on 25 February 2024 to mitigate the impact of database updates. None of the authors had access to any information that could identify individual participants either during or after data collection.

### 2.2 Search strategy

**Figure 1:**
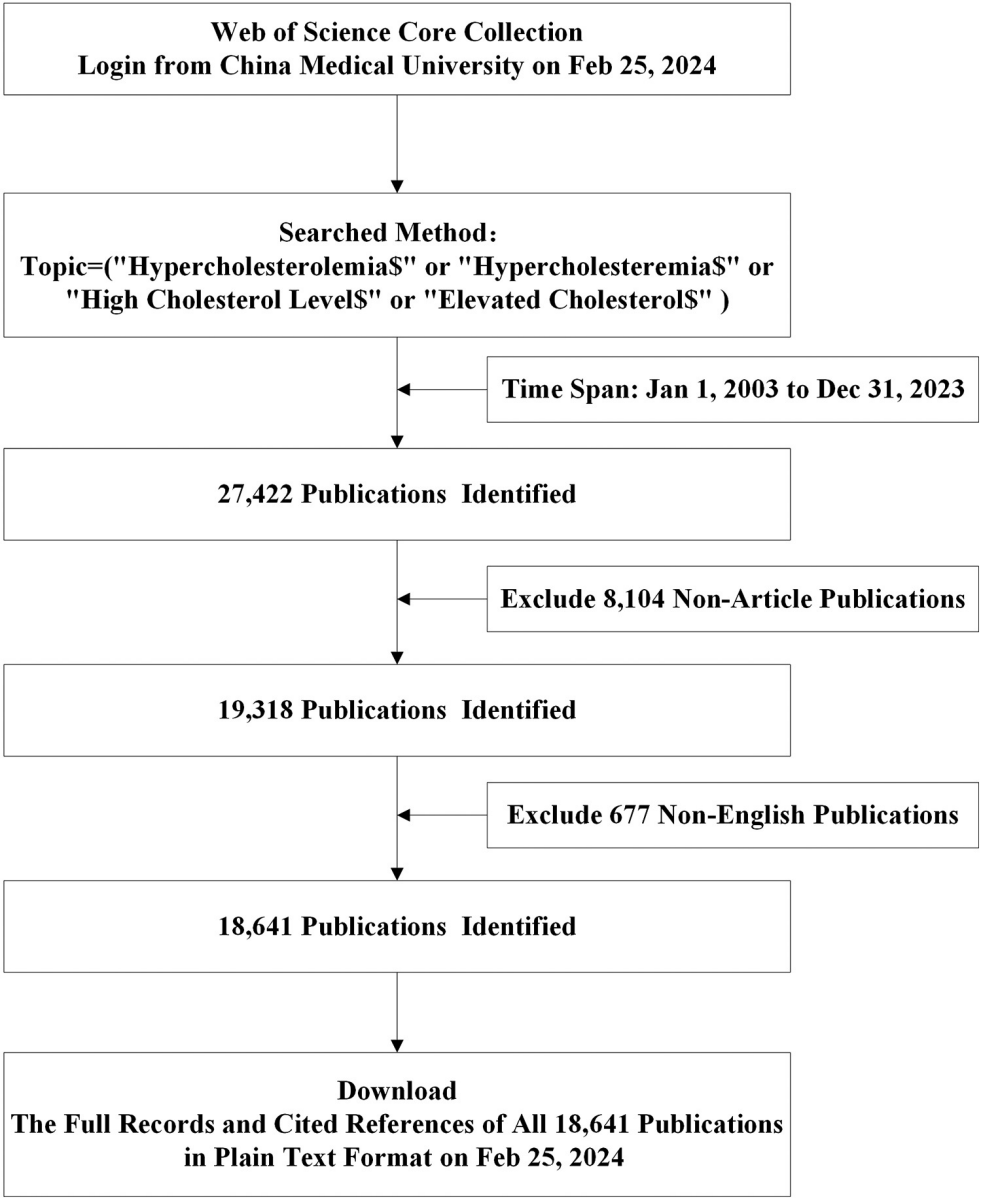
Flow charts of search strategies and article selection methods according to the PRISMA-ScR guidelines.

Our data collection strategy utilized the following search strategy: Topic = (“Hypercholesterolemia$” or “Hypercholesteremia$” or “High Cholesterol Level$” or “Elevated Cholesterol$”). We restricted publications to those in “English”, categorized as “Article”, spanning from 1 January 2003 to 31 December 2023. The search criteria yielded 18,641 studies (Figure 1). The “Full Record and Cited References” for these 18,641 publications were downloaded in “Plain Text” format, which constituted the final dataset.

### 2.3 Software application and visual analysis

VOSviewer is a classical bibliometric analysis software tool designed to analyse academic records and generate network-based maps. It is widely used in literature analysis and research. All valid data downloaded from the WoSCC database were imported into VOSviewer (version 1.6.18) for visual analysis. This included a citation analysis of references, co-authorship analysis of institutions and authors, and co-occurrence analysis of keywords, leading to the construction of overlay visualization maps. Furthermore, a descriptive analysis of the publication year, journal, country, institution, and author was conducted.

## 3 Results

### 3.1 Analysis of publications and citation counts

**Figure 2:**
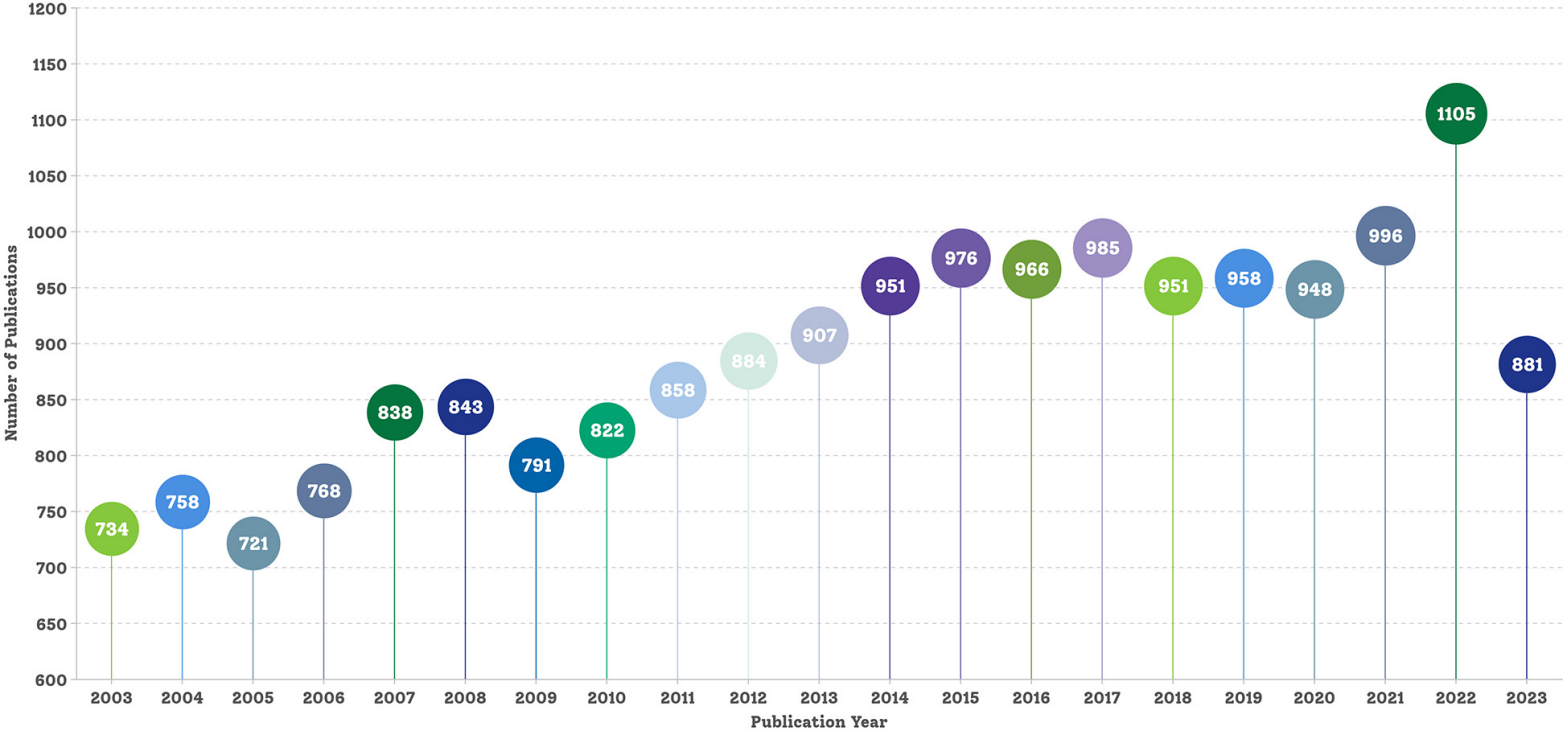
Publication trends in the research field of hypercholesterolaemia.

The final dataset comprised 18,641 bibliographic records of English articles pertaining to hypercholesterolemia published before 31 December 2023. Figure 2 shows the annual publication counts. The data for 2024 are incomplete annual data; therefore, they are not shown in Figure 2. In 2003, there were 734 publications; by 2007, this number surpassed 800 for the first time, reaching 838 articles. Subsequently, in 2013, the number of publications exceeded 900 for the first time, totalling 907. By 2022, the number soared to an all-time high of 1,105, marking a notable 50.54% increase from the 2003 annual publication count. Over the past 20 years, the annual volume of hypercholesterolemia research publications has exhibited an upward trend with slight fluctuations. This upward trend declined slightly from 2023, to 881 publications.

### 3.2 Distribution of source journals

A total of 2,738 academic journals featured publications on hypercholesterolemia, with *Atherosclerosis* (697 articles) ranking first, followed by the *Journal of Clinical Lipidology* (393 articles), and *PloS One* (354 articles). The threshold for journal publication volume was set at 30, and 92 high-impact journals were selected. The selected 92 journals collectively published 7,308 articles, constituting 39.20% of the total dataset. A significant proportion of hypercholesterolemia-related publications exhibit limited citation impact and are dispersed across a wide array of journals. Specifically, among the 667 journals analysed, the average citation remained below 5, encompassing 1,562 relevant publications. Furthermore, 4,081 publications appeared in 2,035 journals, with each journal contributing no more than five papers to this field.

**Figure 3:**
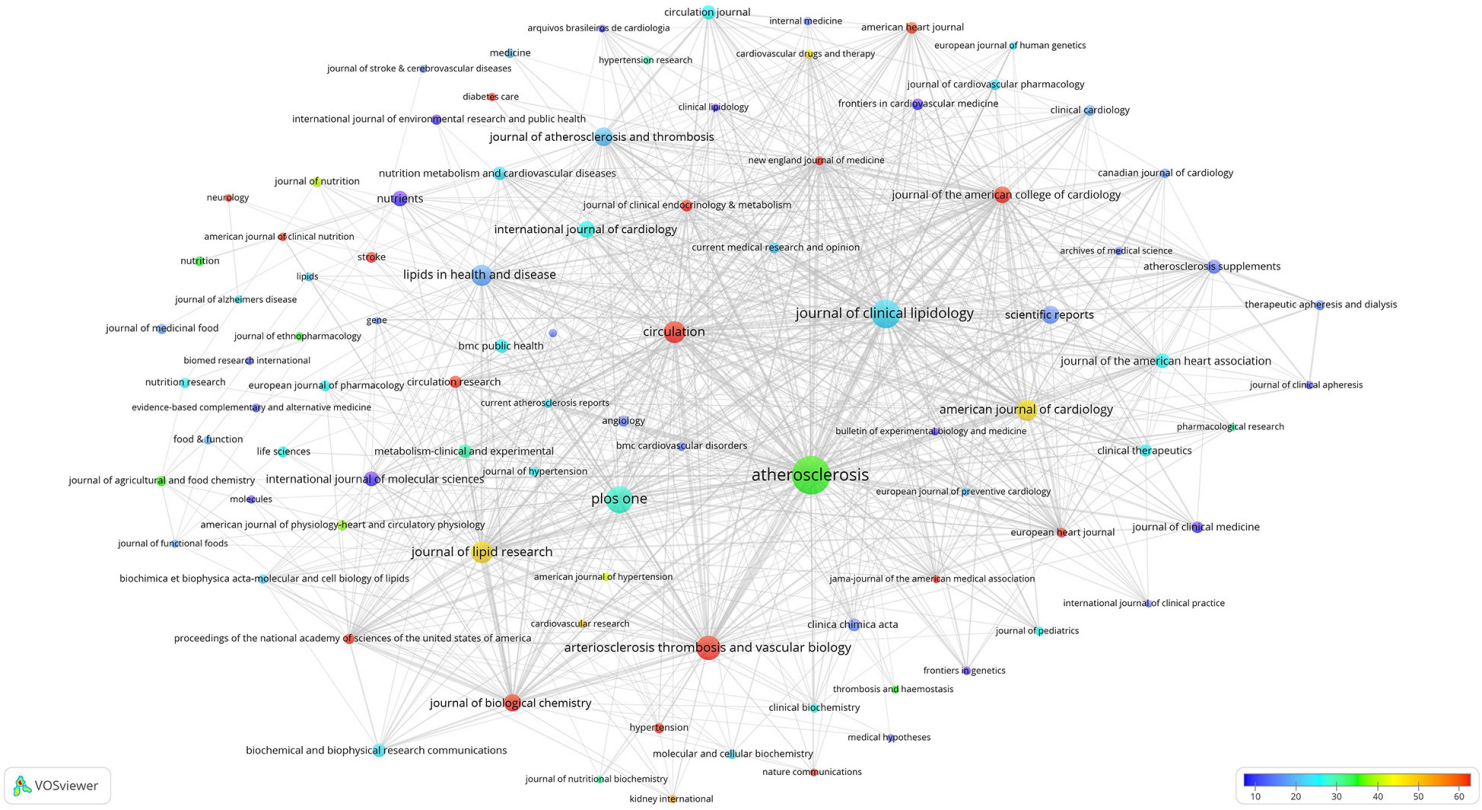
Overlay visualisation map of the citation analysis of 92 high-impact journals. Circle sizes indicate the number of publications, and different colours indicate different average citation counts.

Citation analysis of these 92 high-impact journals was conducted, leading to the construction of the overlay visualization map depicted in Figure 3. The interconnecting lines between different journals represent citation relationships, with thicker lines indicating more citations. The circle size reflects the publication count, whereas the colour gradient (from blue to red) signifies the average citation count from low to high. Among these, 16 journals (highlighted in red) garnered over 60 citations, on average. Notably, the *New England Journal of Medicine* had an average citation count of 750 citations, followed by the *Journal of the American Medical Association* (JAMA; 445 citations) and *Proceedings of the National Academy of Sciences of the United States of America* (PNAS; 222 citations).

### 3.3 Contributions of countries/regions

Our data revealed that 152 countries/regions contributed articles on hypercholesterolemia, with 39 of them surpassing 100 publications and 6 exceeding 1,000 publications. Table 1 lists the top 20 countries/regions based on the number of publications. The United States of America ranked first in terms of publications and citations (5,498 counts, 314,899 citations), followed by China (2,003 counts, 40,741 citations), and Japan (1,481 counts, 43,353 citations). Among the top 20 countries, Sweden had the highest average citation count (*n* = 85). Conversely, China (2,016.74) had the highest average publication year, followed by Poland (2,015.67). The average publication year was calculated by summing the years (as whole numbers) of all publications and then dividing it by the number of publications to obtain the mean. The higher the average publication year, the closer the average time of all article publications to the present.

**Table 1:** Top 20 countries/regions in terms of publication count.

Rank	Country/region	Counts	Citations	Avg. Citations	Avg. Pub. Year
1	USA	5,498	314,899	57	2012.84
2	China	2,003	40,741	20	2016.74
3	Japan	1,481	43,353	29	2011.92
4	Italy	1,286	55,889	43	2014.14
5	Germany	1,043	57,825	55	2013.39
6	Canada	1,003	55,687	56	2013.40
7	England	943	63,793	68	2014.02
8	Netherlands	939	58,353	62	2013.62
9	Spain	889	36,423	41	2014.47
10	France	770	50,859	66	2013.83
11	South Korea	759	17,837	24	2015.29
12	Brazil	629	13,748	22	2015.13
13	Australia	531	35,165	66	2014.55
14	India	521	11,329	22	2014.38
15	Poland	437	11,441	26	2015.67
16	Turkey	416	15,520	37	2012.04
17	Taiwan (China)	390	10,747	28	2013.32
18	Sweden	361	30,809	85	2013.42
19	Greece	294	14,474	49	2012.66
20	Switzerland	294	21,396	73	2014.08

### 3.4 Distribution characteristics of institutions

A total of 15,982 institutions contributed to publications in this field before 2024. Table 2 presents the top 20 institutions based on the publication count, half of which are in the USA. The leading contributors were Harvard University (482 publications), the University of Amsterdam (295 publications), the University of São Paulo (239 publications), the University of Washington (234 publications), and the University of Pennsylvania (222 publications). Among the top 20 institutions, Harvard University had the highest citation count (50,473 times), followed by Brigham and Women's Hospital (26,704 times), and the University of Amsterdam (19,823 times). Furthermore, Brigham and Women's Hospital demonstrated the highest average citation count (134), whereas Capital Medical University in Beijing—one of China's foremost medical institutions—recorded the most recent average publication year (2018.23).

**Table 2:** Top 20 institutions in terms of publication count.

Rank	Institution	Counts	Citations	Avg. Citations	Avg. Pub. Year
1	Harvard University (USA)	482	50,473	105	2013.49
2	University of Amsterdam (Netherlands)	295	19,823	67	2012.89
3	University of Sao Paulo (Brazil)	239	5,135	21	2014.97
4	University of Washington (USA)	234	17,363	74	2013.95
5	University of Pennsylvania (USA)	222	17,623	79	2013.87
6	Baylor College of Medicine (USA)	199	16,990	85	2013.24
7	Brigham and Women's Hospital (USA)	199	26,704	134	2012.87
8	University of Milan (Italy)	197	15,248	77	2016.31
9	Mayo clinic (USA)	196	10,473	53	2013.90
10	University of Montreal (Canada)	189	8,577	45	2015.34
11	Johns Hopkins University (USA)	180	14,632	81	2014.73
12	University of Toronto (Canada)	173	10,613	61	2013.93
13	McGill University (Canada)	161	7,824	49	2013.64
14	Seoul National University (South Korea)	160	3,580	22	2015.89
15	Capital Medical University (China)	146	1,724	12	2018.23
16	Columbia University in the City of New York (USA)	145	10,340	71	2014.45
17	University of California, Los Angeles (USA)	140	10,538	75	2011.12
18	University of Copenhagen (Denmark)	139	10,496	76	2015.27
19	University of Helsinki (Finland)	136	13,522	99	2012.15
20	University of California, San Francisco (USA)	133	9,436	71	2012.83

**Figure 4:**
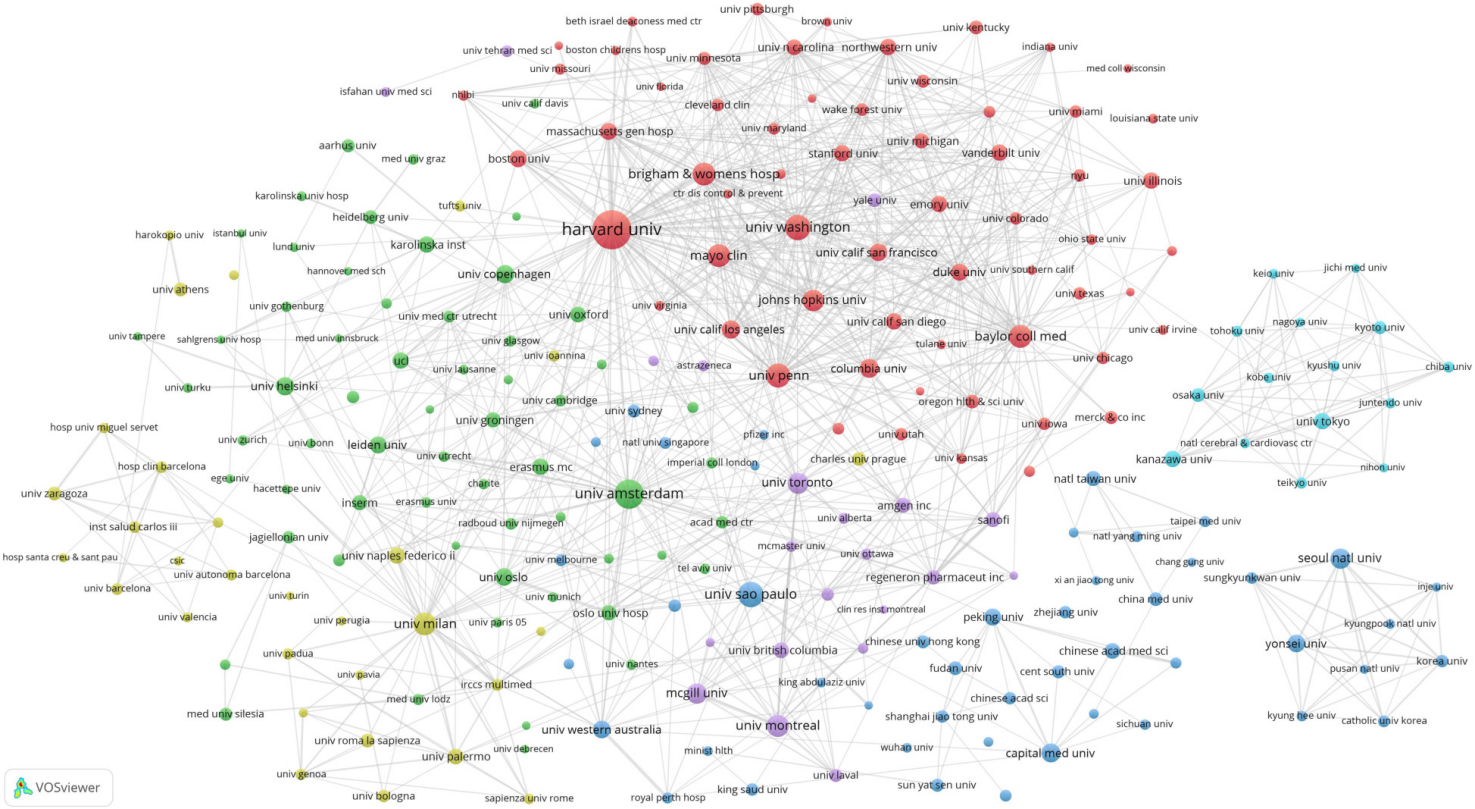
Institutional network map identified nodes and link lines, representing institutions and their collaborative relationships.

We set the threshold for the publication count for institutions at 40 publications and identified 236 highly productive institutions among 15,982 institutions. We used VOSviewer to conduct a co-authorship analysis of 236 highly productive institutions, all of which were in a co-authorship network comprised of six clusters. Figure 4 presents the institutional network map, where nodes denote individual institutions and connecting lines indicate their collaborative links. Figure 4's largest cluster, highlighted in red, comprised 62 institutions. Harvard University was the principal collaborator, partnering with 188 highly productive institutions, followed by the University of Amsterdam, the University of Washington, and the University of Pennsylvania.

### 3.5 Contribution analysis of authors

In total, 103,836 authors contributed to this field since 2003. Table 3 lists the top 20 authors according to the publication count. Kastelein, J.J.P. led with the most publications (121 publications), followed by Watts, G.F. (76 publications) and Santos, R.D. (75 publications). Among the top 20 authors, Kastelein, J.J.P. also had the highest number of citations (11,076 citations), followed by Catapano, A.L. (9,639 citations) and Rader, D.J. (8,823 citations). Conversely, Catapano, A.L. had the highest average citation count (*n* = 140), followed by Rader, D.J. (*n* = 132) and Ballantyne, C.M. (*n* = 104). Among the top 20 authors, Catapano, A.L. had the most recent average publication year (2018.88).

**Table 3:** Top 20 authors in terms of the number of publications.

Rank	Author	Counts	Citations	Avg. Citations	Avg. Pub. Year
1	Kastelein, John J. P.	121	11,076	92	2013.18
2	Watts, Gerald F.	76	6,226	82	2017.86
3	Santos, Raul D.	75	2,534	34	2017.40
4	Hovingh, G. Kees	71	5,084	72	2017.14
5	Catapano, Alberico L.	69	9,639	140	2018.88
6	Rader, Daniel J.	67	8,823	132	2014.66
7	Ballantyne, Christie M.	65	6,777	104	2015.34
8	Kawashiri, Masa-AKI	65	1,235	19	2017.75
9	Tada, Hayato	65	1,154	18	2018.29
10	Harada-Shiba, Mariko	60	1,776	30	2017.55
11	Hegele, Robert A.	60	5,270	88	2017.22
12	Lerman, Lilach O.	60	2,581	43	2010.87
13	Nohara, Atsushi	60	1,502	25	2017.07
14	Civeira, Fernando	59	1,821	31	2015.56
15	Farnier, Michel	58	5,528	95	2017.28
16	Seidah, Nabil G.	58	4,661	80	2013.02
17	Bruckert, Eric	57	4,801	84	2017.00
18	Lerman, Amir	56	2,404	43	2011.27
19	Gaudet, Daniel	52	4,643	89	2016.54
20	Defesche, Joep C.	51	2,316	45	2014.00

Table 4 lists the top 20 authors based on the average number of citations. Sabatine, M.S. had the highest average number of citations (*n* = 479), followed by Giugliano, R.P. (*n* = 348) and Robinson, J.G. (*n* = 308). Among the top 20 authors, Ray, K.K. had the most recent average year of publication (2020.00), followed by Catapano, A.L. (2018.88) and Mach, F. (2018.24).

**Table 4:** Top 20 authors in terms of the average number of citations.

Rank	Author	Counts	Citations	Avg. Citations	Avg. Pub. Year
1	Sabatine, Marc S.	25	11,963	479	2016.88
2	Giugliano, Robert P.	21	7,300	348	2014.95
3	Robinson, Jennifer G.	37	11,384	308	2014.78
4	Chapman, M. John	31	9,127	294	2014.58
5	Wasserman, Scott M.	38	10,026	264	2015.26
6	Koren, Michael j.	20	5,102	255	2015.85
7	Mach, Francois	25	5,551	222	2018.24
8	Schunkert, Heribert	21	4,537	216	2016.67
9	Stein, Evan A.	37	7,503	203	2013.05
10	Scott, Rob	20	3,943	197	2014.20
11	Virani, Salim S.	26	5,115	197	2017.69
12	Nordestgaard, Borge G.	33	6,224	189	2015.79
13	Badimon, Lina	32	6,003	188	2016.25
14	Raal, Frederick J.	44	8,028	182	2017.16
15	Ray, Kausik K.	37	6,436	174	2020.00
16	Pordy, Robert	23	3,956	172	2017.43
17	Laufs, Ulrich	33	5,571	169	2017.94
18	Ginsberg, Henry N.	26	4,063	156	2016.69
19	Kathiresan, Sekar	25	3,601	144	2016.88
20	Catapano, Alberico L.	69	9,639	140	2018.88

**Figure 5:**
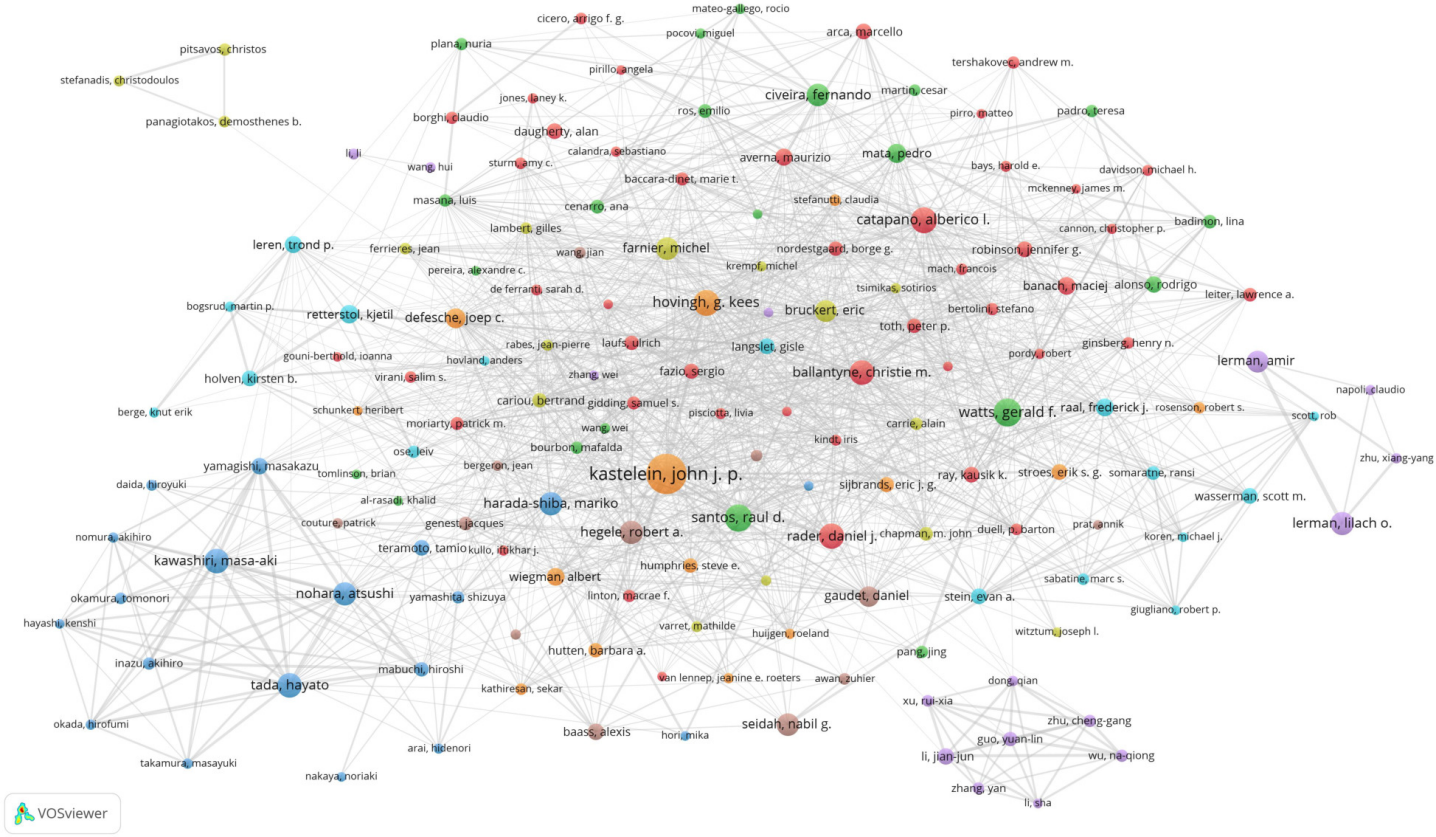
Co-authorship network map identified nodes and link lines, representing authors and their collaborative relationships.

We set the threshold for the number of publications by authors to 20 and identified 167 highly productive authors from a pool of 103,836 authors. We used VOSviewer to conduct a co-authorship analysis of these 167 highly productive authors, 159 of whom formed the largest co-authorship network, consisting of eight clusters. The generated co-authorship network map identifies the nodes and link lines that represent authors and their collaborative relationships. Furthermore, as shown in Figure 5, Kastelein, J.J.P. collaborated with 65 highly productive authors, —more than any other researcher—followed by Hovingh, G.K. (64 highly productive authors) and Santos, R.D (60 highly productive authors).

### 3.6 Keyword analysis

A total of 38,700 keywords were identified in 18,641 publications. By applying a frequency threshold of 50 occurrences, 401 keywords were classified as high-frequency terms. Excluding the search term “hypercholesterolemia”, the top 10 keywords were atherosclerosis (3,252 counts), familial hypercholesterolemia (2,675 counts), cardiovascular disease (2,198 counts), risk (1,952 counts), risk factors (1,806 counts), gene expression (1,717 counts), coronary heart disease (1,689 counts), low-density lipoprotein (1,643 counts), statins (1,473 counts), and prevalence (1,312 counts).

**Figure 6:**
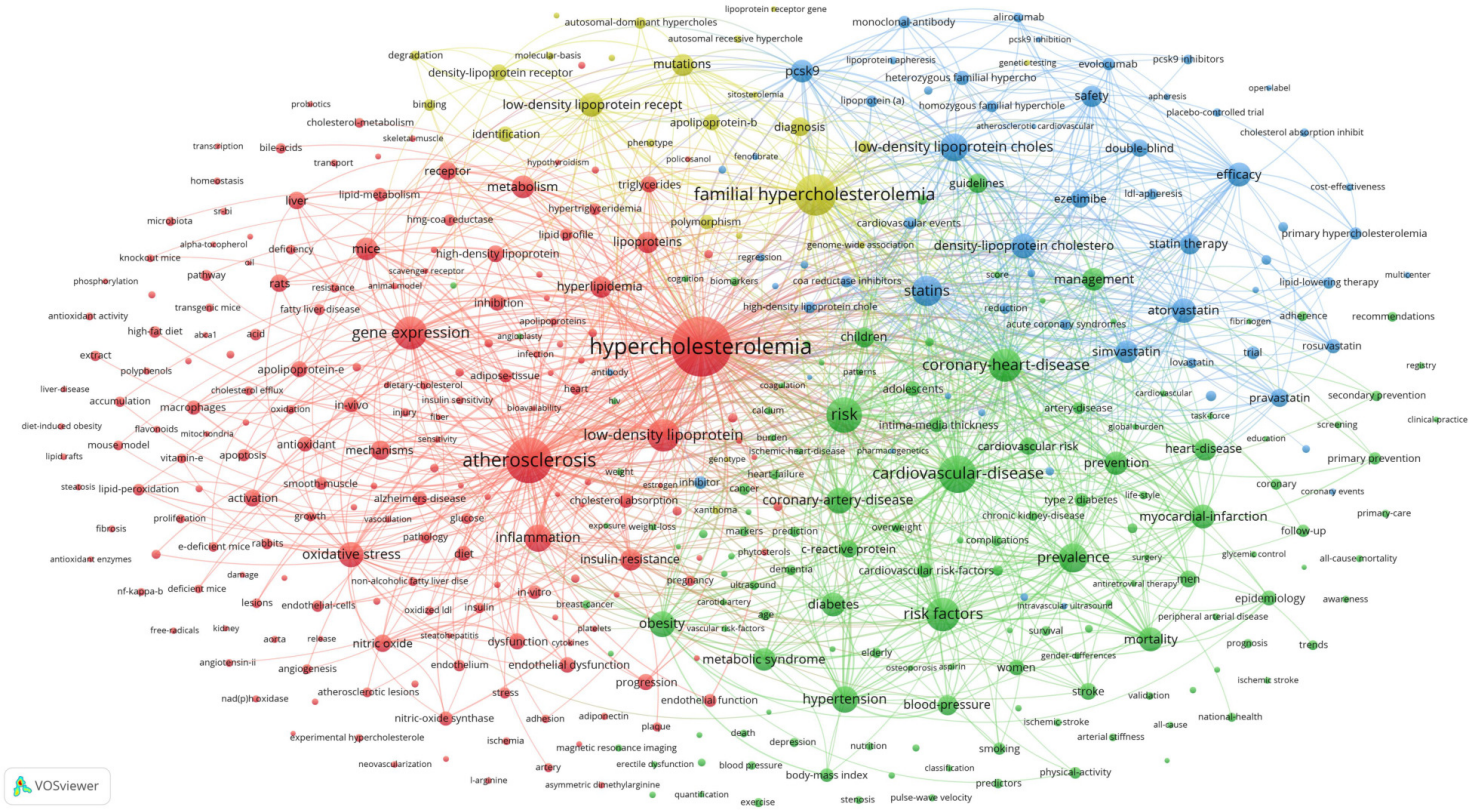
Co-contribution network map identified nodes and link lines, representing keywords and their co-occurrence relationships.

Co-occurrence analysis of these 401 high-frequency keywords was performed using VOSviewer, and a co-contribution network map of the high-frequency keywords was constructed. This analysis formed four clusters represented by different colours (Figure 6). The red cluster comprised 179 keywords and was the largest. According to the characteristics of different clusters, the directions of different clusters were summarized: Cluster 1 (red), Cluster 2 (green), Cluster 3 (blue), and Cluster 4 (yellow) focus on the pathogenic mechanisms; disease prevalence and prevention; drugs and treatments; and familial hypercholesterolemia, respectively.

**Figure 7:**
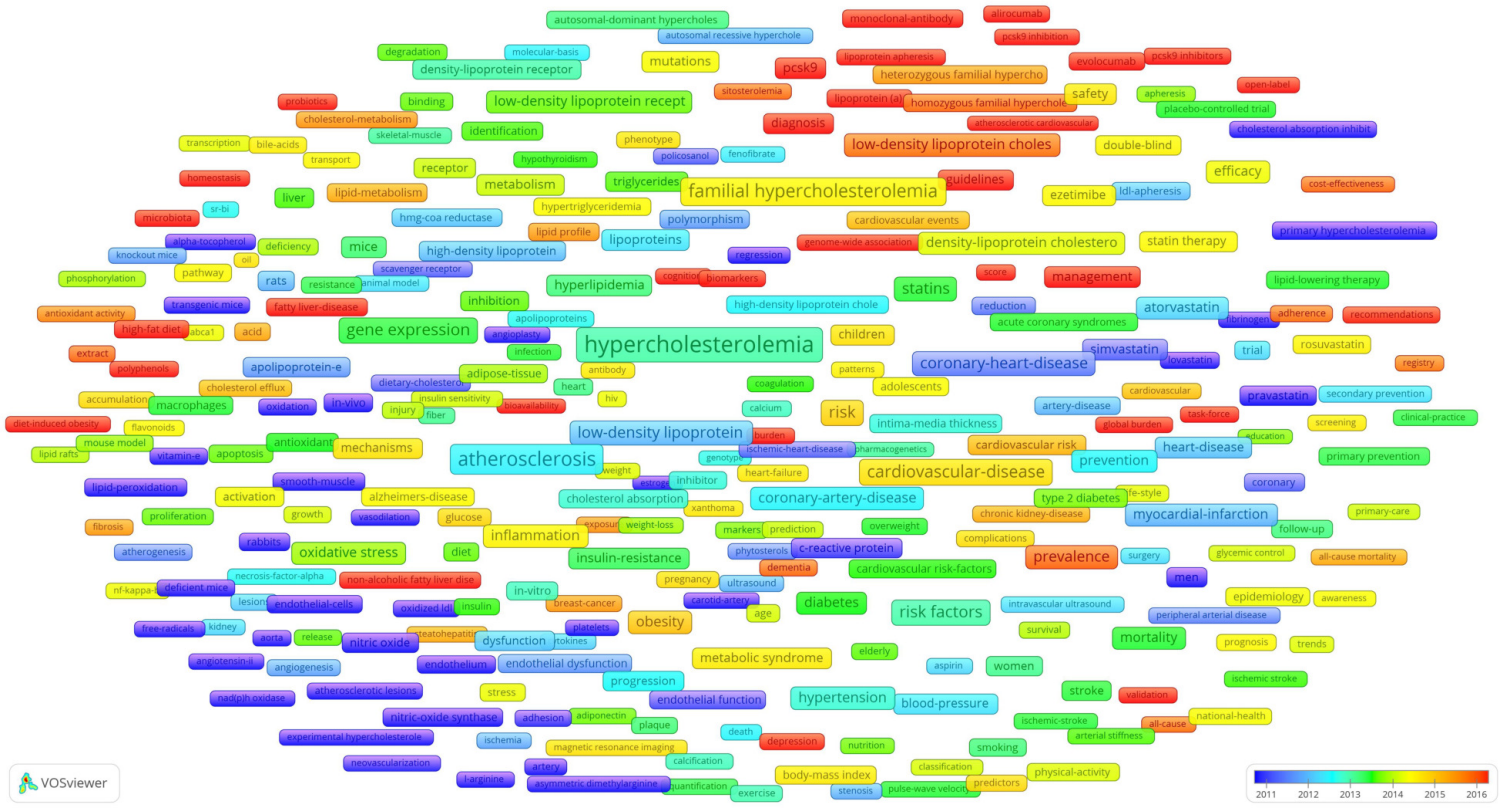
Overlay chart for high-frequency author keywords.

An overlay map of high-frequency author keywords was generated using VOSviewer, with node colours transitioning from blue to red to indicate the average publication year—blue for earlier years and red for more recent ones. The red nodes, representing the latest keywords and emerging hotspots in the research field, are shown in Figure 7. Notably, these keywords had the following average publication years: microbiota (2020.19), atherosclerotic cardiovascular disease (2020.10), pcsk9 inhibitors (2019.62), alirocumab (2018.93), evolocumab (2018.33), pcsk9 inhibition (2018.05), genetic testing (2017.83), non-alcoholic fatty liver disease (2017.49), score (2017.31), burden (2017.30), lipoprotein apheresis (2017.30), probiotics (2017.30), open-label (2017.28), guidelines (2017.21), biomarkers (2017.19), high-fat diet (2016.98), general-population (2016.97), genome-wide association (2,016.89), pcsk9 (2,016.81), lipoprotein (a) (2,016.79), cognitive impairment (2,016.67), variants (2,016.65), diagnosis (2,016.44), global burden (2,016.41), monoclonal-antibody (2,016.38), management (2,016.33), fatty liver-disease (2,016.33), hepatic steatosis (2,016.33), task-force (2,016.28), polyphenols (2,016.22), bioavailability (2,016.16), cognition (2,016.14), diet-induced obesity (2,016.14), validation (2,016.05), and homeostasis (2,016.00).

## 4 Discussion

This study represents the first comprehensive bibliometric analysis of hypercholesterolemia, systematically examining publication counts, high-impact journals, geographic distributions, leading institutions, and prolific authors. Furthermore, it employs VOSviewer (version 1.6.18) to visualize international collaboration networks, delineate research hotspots, and identify emerging topics in the field.

### 4.1 Publication trends

Given that CVD has been the leading cause of mortality and disability worldwide for many years and continues to increase annually, it is not surprising that scientific research on hypercholesterolemia has steadily increased over the past two decades. Moreover, there has been a substantial surge in academic papers in this field, particularly between 2020 and 2022 (Figure 2). This notable increase might be linked to the introduction of novel cholesterol-lowering drugs in clinical practice in recent years. However, whether this surge was related to the coronavirus disease 2019 pandemic remains unclear. The observed decrease in the number of publications for 2023, as depicted in Figure 2, is likely attributable to delays in indexing publications within the Web of Science.

The analysis of journals that published research papers in this domain revealed a substantial concentration of articles published in CVD-focused journals, surpassing those focused-on lipid research (Figure 3). A considerable number of research papers were published in general journals. The journal with the highest average citation counts within this field were those with the highest impact in general, mainly because of the high impact of the journal itself. This observation underscores the importance of publishing in high-impact journals.

In terms of the number of published articles, the United States of America ranked first in terms of the number of articles and citations, significantly surpassing the other countries (Table 1). This may stem from the prolonged emphasis of the United States of America on research in this field, particularly on the development and conduct of clinical trials for cholesterol-lowering drugs. Conversely, although China ranked second in terms of the number of published papers, the average publication year corresponded to the most recent year (2,016.12) (Table 1). This indicates a rapid increase in the number of published papers in this field in China in recent years, highlighting China's recent improvement in research attention and achievements in this field.

### 4.2 International cooperation

According to the statistics of institutions and authors based on the number of published articles, half of the top 20 institutions with the highest article counts are located in the United States of America (Table 2), aligning with the dominance of the United States in the total publications, as previously mentioned. Despite China ranking second in the total publication count, Capital Medical University is the only Chinese institution in Table 2, securing the 15th position. This suggests the scattering of China's article publishing institutions in this field. Similarly, the absence of Chinese authors among the top 20 authors based on publication count and average citations echoes this problem (Tables 3, 4). Kastelein J.J.P emerged as the most prolific author, achieving the highest number of publications and total citations, whereas the work of authors like Sabatine, M.S. et al. garnered the highest average citations—together positioning them among the field's most influential contributors over the past two decades. The publication year of Ray K.K. was the latest (2,020.00), indicating a rapid surge in the number of publications by this author in recent years. This trend suggests notable and swift advancements in the author's contributions to this field in recent years, warranting considerable attention and expectation. Notably, the inclusion of Catapano, A.L. in both Tables 3, 4 underscores the sustained scholarly productivity and substantial impact of his research contributions, reflecting their methodological rigor and broad relevance to the field.

According to the co-authorship analysis of institutions, Harvard University has collaborative ties with a substantial network of 188 high-yield institutions, positioning itself at the core of global research collaboration (Figure 4). In the institutional network map (Figure 4), all 236 institutions were interlinked within a co-network, forming six clusters. The cooperative relationships within each cluster were close and aligned with the institution's country of origin. In contrast to the four centrally positioned clusters exhibiting strong collaborative linkages, the right-sided clusters represent research networks predominantly anchored in China, Japan, and South Korea. This distribution suggests a potential preference for domestic partnerships over international collaborations with leading global institutions, possibly reflecting geopolitical or organizational alignment in knowledge exchange strategies (12).

According to the results of the co-authorship analysis of authors and the co-authorship network map (Figure 5), 159 out of the 167 highly productive authors constituted the largest co-authorship network, forming 8 clusters. Approximately 95% of these highly productive authors were in the co-authorship network, indicating that there is active collaboration among the highly productive authors. Kastelein, J.J.P., Hovingh, G.K., and Santos, R.D. demonstrated the most prolific collaborative engagement among leading authors, with their cooperative networks spanning multiple productive authors. Nohara, A., Tada, H., Kawashiri, M., Yamagishi, M., Mabuchi, H., and Inazu, A. were among the top 20 authors (Table 3) and showed very close partnerships with each other (Figure 5). Their collaborative publications suggest significant teamwork, both internally and in collaboration with other highly productive authors globally. One possible explanation is that the guidelines produced in Europe, the USA, and Japan involve collaborative networks and generally result in high citation rates. Conversely, Li, J.J., situated as the collaboration centre, formed a close partnership in the relatively separate purple cluster, primarily comprising Chinese authors, as shown at the bottom right of Figure 5. While authors within this cluster exhibited robust intra-cluster collaboration, their engagement with leading international authors remained limited. This pattern—also observed in the three-member team positioned in the upper-left quadrant of Figure 5—suggests potential constraints in fostering cross-border collaborative networks.

### 4.3 Research hotspots and emerging topics

The high-frequency keywords derived from the bibliometric and visual analyses of hypercholesterolemia publications can be broadly classified into the following four research foci or hotspots, represented as clusters in Figure 6: pathogenic mechanisms; disease prevalence and prevention; drugs and treatments; and familial hypercholesterolemia.

#### 4.3.1 Pathogenic mechanisms and drugs and treatments

Hypercholesterolemia arises from multifactorial factors, including a poor lifestyle (e.g., excessive caloric intake and obesity), diseases (e.g., hypothyroidism, kidney disease, and liver disease), drug-related factors (e.g., diuretics, β-blockers, glucocorticoids), and inherited or acquired genetic alterations affecting cholesterol transport, synthesis, or metabolic pathways. Scientific research in this field primarily focuses on in-depth exploration of the biological processes influencing cholesterol levels, including cholesterol absorption and transport, cholesterol synthesis, and cholesterol metabolism. Moreover, the development of most cholesterol-lowering drugs or interventions is rooted in the understanding of these molecular mechanisms, which target specific molecules or pathways. Therefore, these two research hotspots intersected and were integrated to create a single summary.

Cholesterol absorption occurs mainly in the intestine and remains unaffected by the *in vivo* synthesis and metabolism of low-density lipoprotein. The process is mainly mediated by the Niemann-Pick C1-Like1 (NPC1L1) protein expressed in the intestine, which regulates circulating LDL-C levels by restricting intestinal cholesterol absorption (13). Ezetimibe, a drug designed to target this pathway, has shown potency as a sterol absorption inhibitor in clinical trials. It selectively blocks biliary and dietary cholesterol absorption in the small intestine by inhibiting NPC1L1 (13, 14). Moreover, studies have confirmed that ezetimibe does not increase LDL receptor activity while reducing LDL-C (15).

Unlike cholesterol absorption, LDL-C biosynthesis occurs in the liver through complex biological processes involving numerous intricate biochemical reactions. Current research on hypercholesterolemia focuses on several key molecules that affect LDL-C synthesis, including β-hydroxy-β-methylglutaryl-CoA (HMG-CoA) reductase, ATP citrate lyase (ACLY), apolipoprotein B (ApoB), microsomal triglyceride transfer protein (MTP), cholesterol ester transfer protein, lipoprotein lipase, angiopoietin-like 3 (ANGPTL3), and ApoCIII. Drugs that inhibit or decrease cholesterol synthesis primarily target these key molecules (Table 5). Examples include statins targeting HMG-CoA, nexletol targeting ACLY, mipomersen targeting ApoB, lomitapide targeting MTP, and evinacumab and vupanorsen targeting ANGPTL3.

**Table 5:** Approved cholesterol-lowering drugs and their targeting molecules involved in the cholesterol synthesis.

Key molecule	Representative drug	Drug function	References
HMG-COAR	Statins	HMG-CoA Inhibitor	(47–49)
ACLY	Nexletol	ACLY inhibitor	(50, 51)
ApoB	Mipomersen	ApoB silencing	(52)
MTP	Lomitapide	MTP inhibitor	(53)
ANGPTL3	Evinacumab	ANGPTL3 silencing	(18)
	Vupanorsen		(54)

The metabolism or clearance of cholesterol has been a hotspot of research for decades since the discovery of the proprotein convertase subtilisin/kexin type 9 (PCSK9) and its direct role in LDL receptor degradation (16, 17). To date, several PCSK9 inhibitors have been approved for clinical use to lower LDL-C levels, such as inclisiran, evolocumab, and alirocumab, and all of them have shown promising efficacy in lowering LDL-C, good tolerance, and short-term reductions in cardiovascular event rates (18–23). However, their long-term safety and impact on cardiovascular events require further investigation. In contrast, apoC-III is a small glycoprotein that can inhibit lipoprotein lipase. Volanesorsen, an antisense inhibitor of apoC-III synthesis, also participates in cholesterol metabolism (24, 25).

In addition to the drugs mentioned above, some nutraceuticals, such as red yeast rice, bergamot, berberine, artichokes, soluble fibre, probiotics, phytosterols, and sterols, alone or in combination, have been reported to have significant lipid-lowering effects. They may also exhibit numerous non-lipid-lowering effects, including amelioration of endothelial dysfunction and arterial stiffness, as well as anti-inflammatory and antioxidant properties. However, according to the International Lipid Expert Panel report (2018), evidence on the long-term safety and efficacy of these supplements for the prevention and treatment of CVDs remains insufficient (26). Cicero et al. reported preclinical and clinical evidence supporting the efficacy and safety of some lipid-lowering nutraceuticals (27). However, Barkas et al. reported that although the cholesterol-lowering effects of plant sterols and stanols were confirmed by previous experiments and human studies, their effects on various markers of atherosclerosis remain controversial (28). In addition, there is a consensus that nutraceuticals cannot replace more effective and evidence-based cholesterol-lowering therapies, such as statins, particularly for individuals at high risk of CVD (26–28). However, red yeast rice extracts, among the most effective cholesterol-lowering nutraceuticals, have been suggested to support lifestyle improvements in low-risk patients, including those who are unable to receive statins or other LDL cholesterol-lowering treatments (29).

#### 4.3.2 Disease prevalence and prevention

Research on the prevalence of hypercholesterolemia is also one of the hotspots. Numerous regional studies worldwide, such as those in Japan (30), the United States (31), Africa (32), Ethiopia (33), China (34), and other countries, have reported epidemiological data regarding the prevalence and awareness rates. In summary, the prevalence of primary hypercholesterolemia and mixed dyslipidemia has risen, and the global burden of elevated low-density lipoprotein cholesterol continues to increase, with the highest levels observed in high-income regions such as Central and Eastern Europe. The increasing burden of cardiovascular diseases related to low-density lipoprotein worldwide highlights the necessity of innovative public health strategies and interventions to mitigate these risks.

The other research hotspot of “prevention” refers to “control the cholesterol to prevent cardiovascular diseases”. Hypercholesterolemia is independently associated with an increased cardiovascular mortality rate. Most clinical guidelines recommend that treatment targets focus on LDL-C for dyslipidaemia because LDL-C levels are used as a marker for treatment response across lipid-lowering trials (5). Consequently, the most reliable and effective method for preventing cardiovascular diseases still revolves around managing LDL-C levels using drugs or other therapies supported by clinical trials. Although significant progress has been made in managing hypercholesterolemia and reducing the associated cardiovascular risks, challenges still remain. The stable cholesterol control rate in recent years indicates that the current management methods may be at a standstill. Therefore, ongoing efforts are required to refine and strengthen prevention and treatment approaches for different populations.

#### 4.3.3 Familial hypercholesterolaemia

Familial hypercholesterolaemia was initially defined as an autosomal dominant genetic disorder associated with LDLR gene mutations, but presently, heterozygous FH has become more specifically identified as carrying rare genetic variants classified as “pathogenic” or “likely pathogenic” in LDLR, APOB, or PCSK9 genes, or having the p.(Leu167del) variant in APOE according to the American College of Medical Genetics and Genomics (ACMG) guidelines (35). In cases where a definitive genetic result is not available, a clinical diagnosis of familial hypercholesterolaemia can still be made based on severe hypercholesterolaemia with tendon xanthomas or LDL-C > 6.4 mmol/L in the proband or first-degree relative, coupled with a dominant inherited pattern (35). This clinical diagnosis may sometimes include other monogenic hypercholesterolaemias, such as monogenic recessive hypercholesterolaemia (36) with homozygosity for LDLRAP1 mutations, dysbetalipoproteinaemia (37) with homozygosity for APOE E2/2 genotype, sitosterolaemia (38) with homozygosity or compound heterozygosity for ABCG5 or ABCG8 mutations, lysosomal acid lipase deficiency (39) with homozygosity for LIPA mutations, hyperlipoproteinaemia(a) (40)with a complex genetic effect of mutations and/or nucleotide polymorphisms of both LPA gene alleles, and polygenic hypercholesterolaemia (41, 42) with a hypercholesterolaemia polygenic score >75th percentile of the distribution, among others.

Familial hypercholesterolemia remains a focal point of research due to its dual clinical relevance: its marked propensity to elevate cardiovascular risk and its multifactorial pathogenesis involving key genes and lipid metabolism pathways. These mechanisms provide foundational insights for identifying biomarkers, novel therapeutic targets, and drug development strategies for cardiovascular and lipid disorders.

#### 4.3.4 Emerging topics

In addition to the four research hotspots, the overlay chart for high-frequency author keywords revealed several emerging topics (Figure 7) including relevant diseases, risk factors, treatment options, and guidelines. The most temporally proximal keywords included microbiota (2,019.76) and PCSK9 inhibitors (2,019.62)—specifically alirocumab (2,018.93) and evolocumab (2,018.33)—reflecting the emerging topics. PCSK9 inhibitors have been at the centre of hypercholesterolemia research for decades and were discussed in the Drugs and Treatments section (4.3.1). Therefore, this section will focus exclusively on microbiota as the sole emerging topic.

Current data indicate that the microbiome extends beyond digestion to encompass several metabolic and inflammatory processes associated with several diseases, including CVDs (43). The metabolites produced by microbes, such as primary and secondary bile acids, trimethylamine N-oxide (TMAO), and short-chain fatty acids, are crucial for maintaining cardiovascular health (44). Individuals with a poor response to rosuvastatin exhibited significantly higher TMAO values than those who received placebo (45). Storm-Larsen et al. performed a retrospective study of patients with familial hypercholesterolemia who had received statins for over 12 months and found that their gut microbial composition, particularly in those taking statins and ezetimibe together, was altered (46). Vourakis suggested several hypothetical mechanisms underlying the effects of probiotics and microbiota composition on cholesterol removal (44). Further research on the relationship between the gut microbiome and cholesterol metabolism is required to deepen our understanding of the microbiome and to advance the development of microbiome-based therapies and precision medicine for patients with hypercholesterolemia and CVD.

### 4.4 Limitations

Similar to other bibliometric studies, our study has some limitations. First, although the Web of Science database contains a vast amount of data, it does not include all relevant articles, which may cause deviations in the results. Second, variations in the authors' and institutions' initials may introduce bias in the statistical results. Finally, only considering English-language publications may have introduced language bias.

Despite the above-mentioned limitations, bibliometric studies aim to provide a comprehensive macroscopic analysis of the development and current state of a research field. This study comprehensively summarizes the research status of hypercholesterolemia by evaluating the prominent keywords, research status, and trends in this field. By offering new and diverging perspectives, our study distinguishes itself from similar previous studies in various respects. For instance, it can assist researchers in identifying core institutions within a field and foster the establishment of novel collaborative relationships. Furthermore, it can enhance readers' comprehension of prevailing trends and trajectories in their areas of interest.

## 5 Conclusion

In recent years, research on hypercholesterolemia has expanded considerably, particularly between 2020 and 2022. The United States had the highest number of publications and many high-producing institutions, while China had recently increased its focus on this field. The main research areas were pathogenic mechanisms, disease prevalence and prevention, drugs and treatments, and familial hypercholesterolemia. Research frontiers included PCSK9 inhibitors and microbiota. Further research is needed on the relationship between the intestinal microbiota and cholesterol metabolism and on the advancement of microbiota therapy and precision medicine.

## Data Availability

The original contributions presented in the study are included in the article/Supplementary Material, further inquiries can be directed to the corresponding authors.
